# Considering autophagy, *β*-Catenin and E-Cadherin as innovative therapy aspects in AML

**DOI:** 10.1038/cddis.2015.314

**Published:** 2015-10-29

**Authors:** K Kühn, W Römer

**Affiliations:** 1 Faculty of Biology, Albert-Ludwigs-University Freiburg, Schänzlestraβe 1, Freiburg 79104, Germany; 2 BIOSS—Centre for Biological Signalling Studies, Albert-Ludwigs-University Freiburg, Schänzlestraβe 18, Freiburg 79104, Germany

Acute myeloid leukemia (AML) is the most common type of leukemia and characterized by a massive accumulation of immature and non-functional myeloid precursor cells in the blood and the bone marrow. A large number of these AML cases involve chromosomal translocations that generate chimeric oncoproteins like AML1-ETO or PML-RAR*α*, which stimulate abnormal proliferation and block myeloid differentiation. As classical chemotherapeutics only tend to highly proliferating cells and do not address at all the issue of differentiation arrest, a better understanding of these cytogenetic aberrations would facilitate the development of targeted therapies. Recently, impressive therapeutic successes have been achieved in acute promyelocytic leukemia (APL) exhibiting striking remission rates and long-term survival up to 90%.^1^ In this, the PML-RAR*α* fusion protein represses the transcription of genes, which are important for myeloid differentiation. The degradation of PML-RAR*α* can be induced by pharmacological doses of all-*trans* retinoic acid (ATRA), thereby enabling transcription and terminal differentiation of immature precursor cells.^2^ However, ATRA is only clinically successful for the small subset of APL patients. Thus, substances that may contribute to the differentiation of other AML subtypes and their modes of action are currently investigated. In a recently published issue of *Cell Death Discovery*, Kühn *et al.*^3^ describe the lectin LecB as inducer of the differentiation of the AML cell line THP-1 and propose a regulation by the interplay between autophagy and *β*-Catenin.

The fucose-binding lectin LecB has been shown to specifically induce differentiation of the THP-1 cell line already after 4 h.^3^ This illustrates a remarkably fast effect as in usual laboratory routine the differentiation of monocytic THP-1 cells to macrophage-like cells is performed by the exposure to phorbol-12-myristate-13-acetate for 72 h.^4^ The effect of LecB is mediated by its binding to glycosylated host cell receptors as blocking of the LecB-binding sites with l-fucose prevented the induction of differentiation. Of note, tumor cells often gain substantial changes in the glycosylation pattern of their surface proteins during cancer progression and metastasis.^5^ Furthermore, it has been shown that LecB is cytotoxic to tumor cells and provokes strong agglutination and an attenuated tumor growth rate.^6^ Further striking roles of lectins have been specified, for example, in bacterial invasion^7^ and the activation of B cells in follicular lymphoma.^8^ In the future, tailor-made engineered lectins^9^ with controlled valency and host cell receptor specificity might become powerful tools as biomarkers and for targeted therapy.

Mechanistically, Kühn *et al.*^3^ elucidated by inhibitor studies that a functional autophagy and a low *β*-Catenin level were essential for the LecB-induced differentiation of THP-1 cells. Interestingly, autophagy is also implicated in ATRA-based differentiation by supporting the degradation of the aberrant fusion proteins and transcription factors.^2^ However, autophagy is so far considered as double-edged sword and its role is not completely clarified as it may act either cytoprotective or cytotoxic to cancer cells, for example, depending on the cell type and the environment. Moreover, an aberrant Wnt/*β*-Catenin signaling, modulated by oncogenic fusion proteins, is frequently implicated in the pathogenesis of leukemia and contributes to stem cell self-renewal, reduced differentiation and apoptosis.^10^ As well, a high *β*-Catenin expression was found in many primary AML samples.^11^ Corresponding to this, Kühn *et al.* demonstrated that a high *β*-Catenin level stabilized proliferation and, remarkably, blocked the initiation of autophagy resulting in low differentiation ability. Surprisingly, the addition of the well-known autophagy-stimulating mTOR inhibitors was not sufficient to induce autophagy as long as the *β*-Catenin level was stable.^3^ Resistances of AML patients to mTOR inhibitors and their low clinical benefit have already been moaned.^12^ On the basis of their observations, Kühn *et al.* proposed that high *β*-Catenin expression was responsible for this resistance, which can be circumvented by the application of LecB. Initiated by the lectin, *β*-Catenin was degraded, autophagy became active and differentiation took place. Moreover, this reduction of the *β*-Catenin level even sensitized THP-1 cells to mTOR inhibitors and enabled a cumulative differentiation-inducing effect (Figure 1). Importantly, these findings implicate novel aspects for the therapy of mTOR inhibitor-resistant AML patients.

However, it has not been addressed so far how the loss of *β*-Catenin occurs. An important regulator of the canonical Wnt/*β*-Catenin signaling pathway is the cell adhesion protein E-Cadherin. In the absence of Wnt ligands, *β*-Catenin is sequestered at the plasma membrane in a complex with E-Cadherin, links it to the actin cytoskeleton and contributes to the regulation of cell–cell contacts. In cancer, *β*-Catenin is often independent on the activation by a Wnt ligand and constitutively active, thereby increasing proliferation and stem cell self-renewal.^10^ The stabilization of *β*-Catenin in a complex with E-Cadherin would therefore decrease these tumor-promoting effects. For this reason, Kühn *et al.* assessed the regulatory potential of E-Cadherin in AML. Indeed, the authors could introduce E-Cadherin as important player in the pathway as its down-modulation by siRNA efficiently attenuated LecB-induced differentiation. In addition, an increased colocalization of E-Cadherin and *β*-Catenin followed by the cellular uptake of these complexes was apparent upon LecB treatment. These findings reveal the crucial role for LecB in the formation of E-Cadherin clusters, which may stabilize *β*-Catenin firmly within these complexes and thereby prevent the nuclear translocation and the target gene expression. This subsequently promotes a cell fate different to that of Wnt-induced *β*-Catenin signaling.^3^

In conclusion, Kühn *et al.* highlight a regulatory role of E-Cadherin for the disruption of the aberrant Wnt/*β*-Catenin signaling pathway. Thereupon, *β*-Catenin degradation facilitates the induction of autophagy and the suppression of deregulated proliferation, which both illustrate crucial aspects of differentiation in AML. Moreover, this study encourages the use of natural and tailor-made synthetic lectins as powerful tools to decipher basic cellular mechanisms and in future therapeutic approaches.

## Figures and Tables

**Figure 1:**
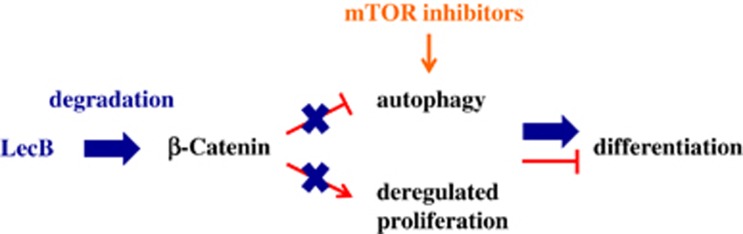
The role of *β*-Catenin during LecB-induced differentiation. A high expression level of *β*-Catenin stimulates proliferation and inhibits autophagy in the AML cell line THP-1 leading to low differentiation ability (shown in red color). Upon LecB treatment, the *β*-Catenin level is reduced, its effects are abolished and differentiation can take place (shown in blue color). In the absence of *β*-Catenin, mTOR inhibitors reinforce the LecB-induced differentiation (shown in orange color)
